# Interactions of commonly used dietary supplements with cardiovascular drugs: a systematic review

**DOI:** 10.1186/2046-4053-1-26

**Published:** 2012-05-31

**Authors:** Salmaan Kanji, Dugald Seely, Fatemeh Yazdi, Jennifer Tetzlaff, Kavita Singh, Alexander Tsertsvadze, Andrea C Tricco, Margaret E Sears, Teik C Ooi, Michele A Turek, Becky Skidmore, Mohammed T Ansari

**Affiliations:** 1 Clinical Epidemiology, The Ottawa Hospital Research Institute and the Department of Pharmacy, The Ottawa Hospital, Ottawa, ON, Canada; 2 Department of Research & Clinical Epidemiology, The Canadian College of Naturopathic Medicine, Toronto, ON, Canada; 3 Ottawa Methods Centre, Clinical Epidemiology Program, Ottawa Hospital Research Institute, University of Ottawa Evidence-based Practice Center, Ottawa, ON, Canada; 4 Li Ka Shing Knowledge Institute, St Michael’s Hospital, Toronto, ON, Canada; 5 Children’s Hospital of Eastern Ontario Research Institute, Ottawa, ON, Canada; 6 Division of Endocrinology and Metabolism, The Ottawa Hospital, University of Ottawa, Ottawa, ON, Canada; 7 Division of Cardiology, The Ottawa Hospital, University of Ottawa, Ottawa, ON, Canada

**Keywords:** Cardiovascular drugs, Dietary supplements, Harms, Systematic review

## Abstract

****Background**:**

The objective of this systematic review was to examine the benefits, harms and pharmacokinetic interactions arising from the co-administration of commonly used dietary supplements with cardiovascular drugs. Many patients on cardiovascular drugs take dietary supplements for presumed benefits and may be at risk for adverse supplement-drug interactions.

****Methods**:**

The Allied and Complementary Medicine Database, the Cochrane Library, EMBASE, International Bibliographic Information on Dietary Supplements and MEDLINE were searched from the inception of the review to October 2011. Grey literature was also reviewed.

Two reviewers independently screened records to identify studies comparing a supplement plus cardiovascular drug(s) with the drug(s) alone. Reviewers extracted data using standardized forms, assessed the study risk of bias, graded the strength of evidence and reported applicability.

**Results:**

Evidence was obtained from 65 randomized clinical trials, 2 controlled clinical trials and 1 observational study. With only a few small studies available per supplement, evidence was insufficient for all predefined gradable clinical efficacy and harms outcomes, such as mortality and serious adverse events. One long-term pragmatic trial showed no benefit from co-administering vitamin E with aspirin on a composite cardiovascular outcome. Evidence for most intermediate outcomes was insufficient or of low strength, suggesting no effect. Incremental benefits were noted for triglyceridemia with omega-3 fatty acid added to statins; and there was an improvement in levels of high-density lipoprotein cholesterol with garlic supplementation when people also consumed nitrates

**Conclusions:**

Evidence of low-strength indicates benefits of omega-3 fatty acids (plus statin, or calcium channel blockers and antiplatelets) and garlic (plus nitrates or warfarin) on triglycerides and HDL-C, respectively. Safety concerns, however, persist.

## **Background**

The American Heart Association estimates that more than 81 million (one in three) American adults have at least one form of cardiovascular disease (CVD) [1]. Pharmaceutical interventions are the front line therapies for the prevention and treatment of CVD in addition to lifestyle and dietary recommendations [2-4].

Billions of dollars are spent annually in the US on complementary and alternative medicine, and a large portion of this expenditure is directly on dietary supplements [5]. Approximately one-third to nearly two-thirds of people experiencing CVD use some form of complementary and alternative medicine that includes dietary supplements, and are thus at risk for potential adverse events from interactions with pharmacologically active agents, and non adherence associated with polypharmacy [6-11]. Evidence of both benefit and harms of adding a supplement to cardiovascular (CV) drugs has been reported [12,13].

While much research is available describing drug-drug interactions in various populations, the evidence is less well described for drug-supplement interactions or simply the effects of add-on supplementation, especially in populations with CVD. The aim of this extensive synthesis review is to examine the evidence for benefits, harms, pharmacokinetic and statistical interactions from co-administration of a set of commonly used dietary supplements with CV drugs.

## **Methods**

We followed a pre-specified and peer-reviewed study protocol. The Agency for Healthcare Research and Quality (AHRQ) commissioned the full evidence report, which is available online [14].

### **Data sources and searches**

Using a peer-reviewed strategy, the Allied and Complementary Medicine Database, the Cochrane Library, EMBASE, International Bibliographic Information on Dietary Supplements and MEDLINE, as well as the grey literature, were searched from study inception until October 2011.

### **Study selection**

A comparative study was eligible if it was published in English or German; other languages were excluded due to concerns with study quality [15] or applicability [16]. We included German language publications given the well-developed regulations for research, practice and use of dietary supplements in Germany [17-19]. We included studies where a dietary supplement was co-administered with a CV drug(s) compared with the drug alone or co-administered with another supplement, and clinical or surrogate CV efficacy or harms, or pharmacokinetic outcomes in any adult population were reported. A dietary supplement was defined as a vitamin, mineral, herb or botanical, amino acid, concentrate or metabolite or extract, enzymes or tissues intended for ingestion in a pill, capsule, tablet, powder or liquid form not represented for use as a conventional food or as the sole item of a meal or diet [20]. We selected to review specific supplements of interest based on reported surveys and input from public and independent technical expert panels [6,21-26]. We aimed to restrict to common supplements and CV medications taken by adults and elderly for which current evidence on possible drug-supplement interaction was lacking. Studies were also included which evaluated the use of coenzyme Q10, *Echinacea*, garlic, ginger, *Ginkgo biloba*, *Panax ginseng,* American ginseng (*P. quinquefolius*), hawthorn, oral magnesium, niacin (≤250 mg/day), omega-3 fatty acids or fish oils, red yeast rice extract, resveratrol, vitamin A, vitamin D with or without calcium, vitamin E or vitamin K as supplements. Finally, we included studies that employed CV drugs commonly used in outpatient settings in the US and Canada. Additional file 1: Table S1.

One reviewer screened all titles and abstracts for potential relevance, and a second verified exclusions at this level. Two independent reviewers assessed the full publication of any potentially relevant studies, with discrepancies resolved by consensus.

### **Data extraction and quality assessment**

Study characteristics, population, intervention, comparator and outcomes data were extracted using standardized forms. The extracted outcomes of interest were categorized into four groups: clinical, intermediate, harms and pharmacokinetic (for example, area under the curve (AUC), half life of CV drug, maximum of peak concentration (Cmax), and amount of time that a drug is present at the maximum concentration (tmax)). The full list of outcomes considered for this review is presented in Additional file 2: Table S2. One reviewer with a clinical background rated study populations with respect to 10-year coronary heart disease (CHD) risk (high, moderate and low) according to the National Cholesterol Education Program Adult Treatment Panel III guidelines [27]. When all participants were healthy non-smokers, study level 10-year CHD risk was categorized as low. We assessed risks of bias according to outcome, using generic items for confounding, selection, performance, detection and attrition bias. Certain quality criteria were specific to particular study designs (for example, allocation generation and concealment applied only to randomized clinical trials (RCTs)). The overall study risk of bias for pre-specified gradable outcomes Additional file 1: Table S1 and Additional file 2: Table S2 was rated as low, moderate or high, and then independently verified. Similarly, the strength of the body of evidence [28] and the applicability of the evidence for outcomes with conclusive results [29] were rated according to the published guidance.

### **Grading the strength of evidence**

A methodologist and a content expert graded the strength of the body of evidence per each outcome (high, moderate, low or insufficient) based on the following four domains: overall risk of bias, consistency, directness and precision [28]. Only a set of pre-specified important outcomes identified *a priori* through consultations with the Technical Expert Panel was graded Additional file 3: Table S3. This was done because customarily only a subset of important outcomes is chosen to grade the strength of evidence - outcomes that are more meaningful for decision-making given a specific research question [28].

The strength of evidence was graded as insufficient when there was no evidence for an outcome, the direction of the estimates was inconsistent across studies, and/or the evidence from the contributing study/studies had an imprecise statistically non-significant pooled estimate (the 95% confidence intervals (CIs) were wide enough to be compatible with either clinical benefit, true no difference or harm).

### **Data synthesis and analysis**

All analyses compared the combination of a dietary supplement plus CV drug(s) with CV drug(s) alone or plus placebo or plus another dietary supplement.

The decision to pool individual study results was based on the degree of similarity in methodological and clinical characteristics of studies under consideration. Meta-analysis was considered when studies were randomized trials that included similar populations, compared the same type of dietary supplement and CV medication in the intervention and control groups, and reported the identical outcome measures in the same statistical format (for example, mean difference (MD) or geometric mean ratio (GMR)). The estimates of post-treatment MD for continuous outcomes and relative risk (RR) for binary outcomes (except for rare events) of individual studies were pooled using a random-effects model by DerSimonian and Laird [30]. The choice of this model was based on the assumption that some residual clinical and methodological diversity might still exist across the pooled studies despite the similarities [31]. For dichotomous outcomes with event rates < 1%, the Peto odds ratios (ORs) based on a fixed effects model were calculated. The results from studies with zero events in one of the arms were pooled using the fixed effects Mantel-Haenszel method without continuity correction. Studies with zero events in both arms were not pooled [31].

The degree of statistical heterogeneity across pooled studies was assessed by visual inspection of the forest plot and the generation of Cochran’s Q (α = 0.10) and the I^2^ statistic. We planned to explore clinical (for example, gender, age, ethnicity) and methodological factors (for example, parallel versus crossover design, risk of bias, type of analysis, baseline health status) as potential sources of statistically significant heterogeneity (Chi-square statistic *P* < 0.10 and/or I^2^ > 50%).

We followed the US Food and Drug Administration (FDA) guidance for analysis and interpretation of drug interaction pharmacokinetic studies; the two comparators are considered bioequivalent (no clinically important difference) if the lower and upper bounds of the 90% CI for the GMR were between 0.8 and 1.25 [32]. Where applicable, we examined statistical interactions between supplements and drugs by calculating the synergy index [33].

All analyses were performed using Comprehensive Meta Analysis version 2.2.057, StatsDirect statistical software and R: A Language and Environment for Statistical Computing, Foundation for Statistical Computing [34-36].

### **Role of the funding source**

The AHRQ supported this study but had no role in formulating study questions, conducting the systematic review, or approving the manuscript for submission and publication.

## **Results**

**Figure 1:**
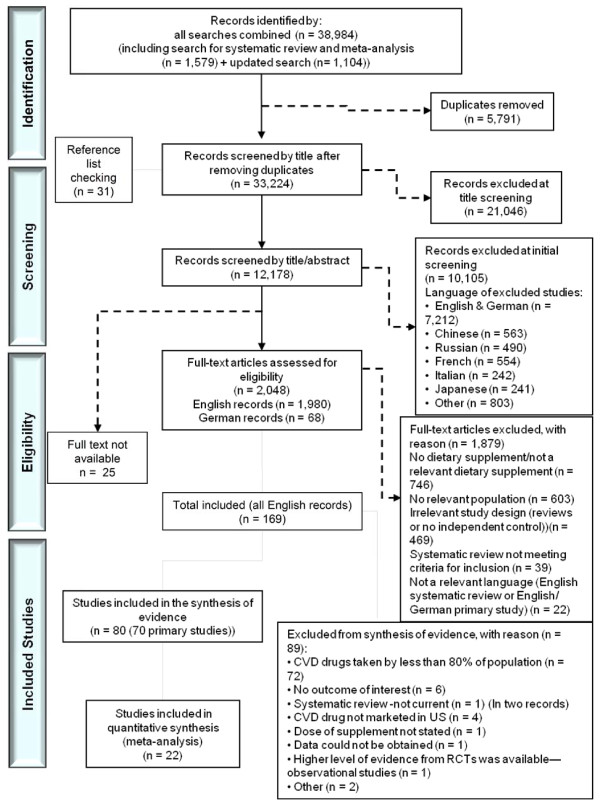
**Preferred Reporting Items for Systematic Reviews and Meta-Analyses (PRISMA). **Summary of evidence search and selection.

In total, 32,314 records were screened for eligibility (Figure 1). Sixty-three unique English language studies, including one of observational design, contributed evidence to the present review regarding the interactions of supplement-drug combinations [37-99]. We found no relevant unique German publications, nor a good quality systematic review to obviate the need for *de novo* synthesis of evidence. Most studies had a moderate risk of bias for the gradable outcomes. A paucity of studies precluded exploration of subgroup heterogeneity and how this may have affected outcomes. Statistical interactions between supplement(s) and drug(s) were generally not reported.

Here, we outline selected results for gradable outcomes Additional file 3: Table S3 from the systematic review [100] limited to American ginseng, coenzyme Q10, *Echinacea*, garlic, *Ginkgo biloba*, omega-3 fatty acids and vitamin E. While the full report commissioned by AHRQ can be found at http://www.effectivehealthcare.ahrq.gov/index.cfm) [101], selected results reported here are limited to those that provide some signal of evident or potential drug interaction.

Overall, evidence for clinical, harms and pharmacokinetic outcomes was inconclusive (grade: insufficient). The majority of the reported evidence was based on intermediate efficacy outcomes (grade: insufficient to low).

These results represent evidence on: clinical outcomes of effectiveness from 19 RCTs [37-55] (Table 1); intermediate surrogate outcomes from 52 RCTs and two non-RCTs [38,39,41,43,45,48-94] (Tables 2 and 3); harms identified from 52 RCTs and a single retrospective cohort study [37-41,43,45-48,50-68,70-73,76-78,80-84,87,90-99] (data available online[100]); and lastly direct evidence of pharmacokinetic data on interactions between supplements and CV drugs from 11 RCTs [37,60,63,66,71,83,96,98](data available online [100]).

**Table 1:** **Gradable clinical outcomes for dietary supplements plus cardiovascular drugs**^**a**^

**Item**	**Supplement (dose)**	**Cardiovascular drug**	**Number of studies, sample size, characteristic (treatment duration)**	**Outcome (combination versus control)**
**Clinical outcome efficacy**				
**All-cause mortality**	Coenzyme Q10 (100 mg/day)	ACE inhibitors (80% of participants were also taking digoxin, furosemide, hydralazine and/or nitrates)	Single study [53]; 30 mostly male patients with left ventricular dysfunction (3 months)	Death: none versus one
	*Ginkgo biloba* (40 mg four times a day)	Antiplatelet agents	Single study [47]; 62 South Asian patients with previous ischemic stroke (1 month)	No deaths
	Omega-3 fatty acids (4 g/day)	Aspirin	Single study [46]; 291 patients admitted for coronary artery bypass grafting (1 year)	Death: five versus four
		Statins	Single study [37]; 50 healthy nonsmoking adults (2 weeks)	No deaths
		Warfarin	Single study [46]; 319 patients admitted for coronary artery bypass grafting (1 year)	Deaths: three versus two
		Fenofibrate	Single study [55]; 167 participants with hyperglycemia (2 months)	No deaths
**Quality of life**	Coenzyme Q10 (100 mg/day)	ACE inhibitors	Single study [53]; 30 mostly male patients with left ventricular dysfunction (3 months)	Minnesota ‘Living with Heart Failure’ questionnaire (mean sum of all scores post-treatment 26.7 ± 17.9 versus 26.5 ± 18.7
**Myocardial infarction**	Omega-3 fatty acids (1.8 g eicosapentaenoic acid + 1.2 g docosahexaenoic acid)	Aspirin + calcium channel antagonists	Single study [50]; 58 patients who had undergone successful coronary angioplasty (6 months)	Acute MI: 4 versus 2
				RR 1.70 (95% CI 0.32, 8.84)
**Arrhythmia**	Omega-3 fatty acids (4 g/day)	Statins	Single study [41]; 256 patients with persistent hypertriglyceridemia despite statin therapy (2 months)	Arrhythmia: one versus none
**Stroke**	Vitamin E (0.4 g/day)	Aspirin	Single study [48]; 100 patients with previous reversible or irreversible ischemic neurologic deficit (2 years)	Fatal or non-fatal stroke: three versus six
**Ischemic stroke, hemorrhagic stroke and transient ischemic attack**	Vitamin E (600 IU/day)	Aspirin	Single study [40]; 19,934 healthy women (10 years)	Composite outcome of nonfatal MI, nonfatal stroke and vascular death, RR 0.95 (95% CI 0.79, 1.13)

^a^Inconclusive results: studies had an imprecise statistically non-significant pooled estimate (the 95% CIs were wide enough to be compatible with either clinical benefit, true no difference or harm). *ACE*: angiotensin-converting enzyme; *CI*: confidence interval; *MI*: myocardial infarction; *RR*: relative risk.

**Table 2:** Gradable intermediate outcomes for dietary supplements plus cardiovascular drugs (low grade evidence)

**Outcome measures**	**Dietary supplement**	**Cardiovascular drug(s)**	**Conclusion effect estimate**	**Applicability**
**Lipid profile**	Co-Q10 (200 mg/day)	Fenofibrate	No difference for HDL-C (one study): MD,1.55 mg/dL (95% CI −6.78, 3.68)	Mean age:
				53 years
				Mixed gender
				High CHD risk
				12 weeks treatment
**Lipid profile**	Garlic (4 g/day)	Nitrates	**In favor of combination**	Unknown age, gender
			HDL-C (one study): MD, 8.40 mg/dL (95% CI 1.91, 14.89)	High CHD risk
				12 weeks treatment
**Lipid profile**	Omega-3-fish oil (3.6 g/day omega-3 to 9.2 g/day fish oil)	Statins	**In favor of combination**	Mean age: 45 to 63 years
			TG (two studies pooled): MD, -74.95 mg/dL (95% CI −95.80, -54.10)^a^	Mixed CHD risk
			**No difference between combination and CV drug alone**	Mixed gender
			HDL-C (six studies pooled): MD, 2.26 mg/dL (95% CI −1.8, 6.3)	Up to 25 weeks treatment
			LDL-C (five studies pooled): MD, 1.3 mg/dL (95% CI −3.6, 6.2)	
			Achieving LDL-C targets: RR 0.93 (95% CI 0.84, 1.03)	
			Achieving HDL-C targets (one study): and 1.00 (95% CI 0.90, 1.10)	
**Lipid profile**	Omega-3-fish oil (1.8 g/day)+	Calcium channel blockers + aspirin	**In favor of combination**	Mean age: 57 y;
			TG (two studies not pooled): MD −81.00 mg/dL (95% CI −125.30, -36.70) and MD −54.00 mg/dL (95% CI −94.1, -13.90)	85% men
				High CHD risk
				Up to 6 weeks treatment
**Lipid profile**	Omega-3-fish oil (3.2 g/day)	Calcium channel blockers + aspirin, or dipyridamole	**In favor of CV drug alone**	Mean age: 56 y;
			LDL-C (one study): MD 21.00 mg/dL (95% CI 3.30, 38.70)	100% men
			**In favor of combination**	High CHD risk
			TG (one study): MD −81.0 mg/dL (95% CI -125.30, -36.70)	
				Up to 12 weeks treatment
**Lipid profile**	Vitamin E (900 mg/day)	Nifedipine	**In favor of combination**	Elderly; mixed gender
			LDL-C (one study): MD −39.83 mg/dL (95% CI −71.29, -8.37)	High CHD risk
				12 weeks treatment
			**In favor of combination**	
			TG (one study): MD, -23.91 mg/dL (95% CI -35.89, -11.93)	
**Blood pressure**	Omega-3-fish oil (2 g/day)+	Statins	**In favor of combination**	Mean age: 44 to 53 y; mixed gender
			Systolic blood pressure (one study): MD, -8.50 mmHg (95% CI -16.3, -0.66)	Mixed CHD risk
				5 weeks treatment
			Systolic blood pressure (one study): median change from baseline −5.0 versus + 0.3 mmHg	
			**No difference between combination and CV drug alone**	
			Diastolic blood pressure (one study): MD, 0.20 mmHg (95% CI -4.76, 5.16)	
	Omega-3-fish oil (4 g/day fish oil)+	Statins	Diastolic blood pressure (one study): Median reductions from baseline -3.30 versus −1.80 to	Mean age: 58 y; Mixed gender
				Unclear CHD risk
				6 weeks treatment

^a^Both studies recruited participant with higher baseline levels of triglyceride (>200 mg/dL). *CHD*: coronary heart disease; *CI*: confidence interval; *CV*: cardiovascular; *HDL-C*: high density lipoprotein-cholesterol; *LDL*-cholesterol: low density lipoprotein-C; *MD*: mean difference (post-treatment values); *RR*: relative risk; *TG*: triglycerides.

**Table 3:** **Gradable intermediate outcomes for dietary supplements plus cardiovascular drugs (insufficient grade evidence)**^**a**^

**Item**	**Supplement (dose)**	**Cardiovascular drug**	**Number of studies, sample size, characteristic (treatment duration)**
**All lipids (Low and high density lipoproteins-cholesterol, triglycerides, total cholesterol)**	Coenzyme Q10 (100 to 200 mg/day)	Statins	Two studies; 49 hypercholesterolemic patients [57], and 44 patients with statin-induced myopathic pain [39]; (12 weeks)
	Coenzyme Q10 (200 mg/day)	Fenofibrate	Participants with type II diabetes and high CHD risk
	Garlic (4 g/day)	Warfarin	Single study [59]; 48 participants with prosthetic heart valves, or diagnosed with deep vein thrombosis, valvular heart disease or atrial fibrillation
	Garlic (4 mL/day)	Statins + aspirin	Single study [62]; 23 participants with, or at high risk for, coronary artery disease (1 year)
	Garlic (4 g/day)	Nitrates	Single study [61]; 60 participants with coronary
			artery disease (1 year)
	*Ginkgo biloba* (120 mg/day)	Aspirin	Single study [64]; 50 young healthy men (1 week)
	Omega-3 fatty acids (4 g/day)	Fenofibrate	Single study [55]; 167 participants with unclear CHD risk (8 weeks)
	Omega-3 fatty acids (3 g/day)	Calcium channel blockers	Single study [49], 22 participants with variant angina (16 weeks)
	Omega-3 fatty acids (4 g/day)	Niacin + aspirin	Single study [77]; 14 participants with atherogenic dyslipidemia (12 weeks)
	Omega-3 fatty acids (10 g/day)	Aspirin	Two studies [80,84]; 30 healthy participants (2 to 3 weeks)
	Vitamin E (0.6/day)	Gemfibrozil	Single study [88]; 67 participants with hyperlipidemia (4 weeks)
	Vitamin E (100 mg/day, 100 IU/day)	Statins	Pooled results for four studies[89,91-93]; 192 highly selective participants (24 weeks)
**Triglycerides**	Omega-3 fatty acids (4 g/day)	ACE inhibitors	Two studies [78,79]; 58 participant with renal dysfunction or hypertension (6 to 25 weeks)
	Omega-3 fatty acids (4 to 9 g/day)	Statins	Three studies [38,72,82]; 420 highly selected participants with low or unclear CHD risk (4 to 18 weeks)
	Vitamin E (900 mg/day)	Antiplatelet agents	Single study [86]; 16 participants with high CHD risk (6 weeks)
**Low density lipoprotein-cholesterol**	Omega-3-fish oil (1.8 g/day)	Calcium channel blockers + aspirin	Single study [50]; 107 participants with pre-coronary angioplasty (6 weeks)
**High density lipoprotein-cholesterol**	Vitamin E (900 mg/day)	Nifedipine	Single study [94]; 30 participants with high CHD risk (16 weeks)
	Omega-3 fatty acids (1.8 g/day)	Calcium channel blockers + aspirin	Single study [50]; 107 participants with pre-coronary angioplasty (6 weeks)
	Omega-3 fatty acids (3.2 g/day)	Calcium channel blockers + aspirin + dipyridamole	Single study [51]; 82 participants with post-coronary angioplasty (12 weeks)
**Blood pressure (systolic and diastolic)**	Coenzyme Q10 (200 mg/day)	Fenofibrate	Single study[56]; 80 participants with type II diabetes and high CHD risk (12 weeks)
	Garlic (4 g/day)	Warfarin	Single study [59]; 48 participants with prosthetic heart valves, or diagnosed with deep vein thrombosis, valvular heart disease or atrial fibrillation
	*Ginkgo biloba* (120 mg/day)	Aspirin	Single study [64]; 50 young healthy male volunteers (1 week)
	*Ginkgo biloba* (120 mg/day)	Cilostazol	Single study [67]; 10 healthy South Asian men (1 day)
	Omega-3 fatty acids (10 g/day)	Aspirin	Two studies [80,84]; 30 healthy participants (2 to 3 weeks)
	Omega-3 fatty acids (4 g/day)	Beta-adrenergic antagonists	Single study [85]; 25 participants with unclear CHD risk (6 weeks)
	Vitamin E (600 mg/day)	Furosemide	Single study [87]; 24 hypertensive participants (4 weeks)
		Gemfibrozil	Single study [88]; 67 participants with hyperlipidemia (4 weeks)
	Vitamin E (900 mg/day)	Nifedipine	Single study [94]; 30 participants with high CHD risk (16 weeks)
**International normalized ratio**	*Echinacea* (5 g/day)	Warfarin	Single study [97]; 12 healthy volunteers (2 weeks)
	Garlic (4 g/day)		Two studies; 48 participants with high CHD risk [59](12 weeks), and 16 healthy men with known CYP2C9 and VKORC1 genotype [60] (2 weeks)
	Ginger (3.6 g/day)		Single study [63];12 healthy male volunteers (7 days)
	*Ginkgo biloba* (12 g/day)		
	*Panax ginseng* (1.5-2 g/day)		Two studies; seven healthy men [70] (1 week), 25 patients with ischemic stroke [68] (2 weeks)
	Omega-3-fish oil (4 mg/day)		Single study [54]; 11 participants with unclear CHD risk (4 weeks)

^a^Inconclusive results: studies had an imprecise statistically non-significant pooled estimate (the 95% CIs were wide enough to be compatible with either clinical benefit, true no difference or harm). ACE: angiotensin-converting enzyme; CHD: coronary heart disease.

Overall, the majority of included RCTs were of moderate risk of bias with only 25% of the studies explicitly reporting adequate sequence generation and 9% reporting allocation concealment. Adherence to CV medication was reported for co-administration of coenzyme Q10 plus statins, omega-3 fatty acids plus statins, and vitamin E plus statins in five studies [38-40,43,52].

### **American ginseng (*panax quinquefolius)***

#### ***Clinical outcome efficacy***

No evidence.

#### ***Intermediate outcome efficacy or harms***

Evidence from three RCTs for co-administration of ginseng and warfarin was inconclusive (grade: insufficient) [68-70].

#### ***Pharmacokinetic outcomes***

American ginseng (2 g/day from weeks 2 to 4) caused a statistically significant reduction in the warfarin AUC (between group difference in median change from week 1 to week 4, -0.64 μg/mL per day (95% CI −1.25, -0.13)) [69]. The clinical significance of this finding is unclear because the analysis was not based on GMRs.

### **Coenzyme Q10**

#### ***Clinical outcome efficacy***

Evidence for the effect of coenzyme Q10 co-administered with angiotensin-converting enzyme inhibitors on all-cause mortality and quality of life in one study of mostly male patients with left ventricular dysfunction was inconclusive (grade: insufficient) [53]. No differences in adherence to statins were noted with co-administration of coenzyme Q10 [39].

#### ***Intermediate outcome efficacy***

Low grade evidence was available from one trial indicating no significant difference in high-density lipoprotein-cholesterol (HDL-C) for combination of coenzyme Q10 plus fenofibrate versus fenofibrate alone [56].

#### ***Harms***

Evidence from five RCTs for the combination of coenzyme Q10 and statins, fenofibrate or angiotensin-converting enzyme inhibitors was inconclusive (grade: insufficient) [39,53,56-58].

#### ***Pharmacokinetic outcomes***

No evidence.

### **Echinacea**

#### ***Clinical outcome efficacy***

No evidence.

#### ***Intermediate outcome efficacy or harms***

Evidence from one study for the combination of *Echinacea* and warfarin was inconclusive (grade: low) [97].

#### ***Pharmacokinetic outcomes***

Results of one study showed no clinically important but a statistically significant decrease (grade: insufficient) in S-warfarin AUC_∞,_ an increase in drug clearance and an apparent increase in volume of distribution (GMRs: 0.92 (90% CI 0.85, 0.99); 1.09 (90% CI 1.01, 1.18); and 1.09 (95% CI 1.03, 1.18), respectively) [97].

### **Garlic**

#### ***Clinical outcome efficacy***

No evidence.

#### ***Intermediate outcome efficacy***

In one study [61], a combination of garlic (4 g/day) and nitrates improved mean levels of HDL-C compared with nitrate alone (grade: low). Three other studies provided insufficient evidence for a combination of garlic plus warfarin, nitrates, or statin in addition to aspirin [60-62].

#### ***Harms***

Evidence in four RCTs for garlic co-administered with warfarin, nitrates, or statins plus aspirin in healthy men [60] or those with CV conditions was inconclusive (grade: insufficient) [59,61,62].

#### ***Pharmacokinetic outcomes***

Evidence from two studies demonstrated neither clinically important (AUC, half-life or clearance; grade: low) [60] nor statistically significant (C_max_; grade: insufficient) [102] interactions between garlic extracts and warfarin.

### **Ginkgo biloba**

#### ***Clinical outcome efficacy***

Insufficient evidence was found for the effect of *Ginkgo biloba* plus antiplatelets (aspirin and/or pentoxifylline) on all-cause mortality (no deaths in either group) in South Asians with previous stroke [47].

#### ***Intermediate outcome efficacy***

Evidence from five RCTs for the combination of *Ginkgo* and acetylsalicylic acid, clopidogrel, ticlopidine, warfarin or cilostazol was inconclusive (grade: insufficient) [63-67].

#### ***Harms***

Seven studies provided insufficient evidence for harm for the co-administration with warfarin, digoxin, aspirin, aspirin and/or pentoxifylline, nitrates, cilostazol or clopidogrel, or ticlopidine [47,63-67,98].

#### ***Pharmacokinetic outcomes***

Three studies examined pharmacokinetic interaction of *Ginkgo biloba* and specific CV drugs (ticlopidine, digoxin and warfarin) [63,66,98]. Study results for clearance, AUC, half-life, or C_max_ were either clinically not important or statistically non-significant (grade: insufficient).

### **Omega-3 fatty acids**

#### ***Clinical outcome efficacy***

Evidence on all-cause mortality for healthy individuals and highly selected patients with dyslipidemia taking statins as an add-on therapy [47], and patients with unclear to high 10-year CHD risk taking aspirin, warfarin or fenofibrate [55], was inconclusive (grade: insufficient). Similarly, evidence on acute myocardial infarction in high-risk patients taking a calcium channel antagonist was inconclusive [50]. Evidence from two studies on the incidence of restenosis following successful coronary angioplasty in patients taking omega-3 fatty acids as an add-on to antiplatelet and calcium channel antagonist therapies was conflicting. One study showed significantly lower rates in the combination group (RR 0.40, 95% CI 0.20, 0.82) [51] and the other study reported no significant difference between the groups (RR 1.33, 95% CI 0.76, 2.30) [50].

There is inconsistent evidence for a benefit when omega-3 fatty acids were added to conventional antiplatelet therapy and calcium channel antagonists on rates of acute myocardial infarction [50,51]. In three short-term RCTs [38,43,52], adherence to CV drugs as judged by pill count was greater than 95% in both treatment groups.

#### ***Intermediate outcome efficacy***

**Figure 2:**
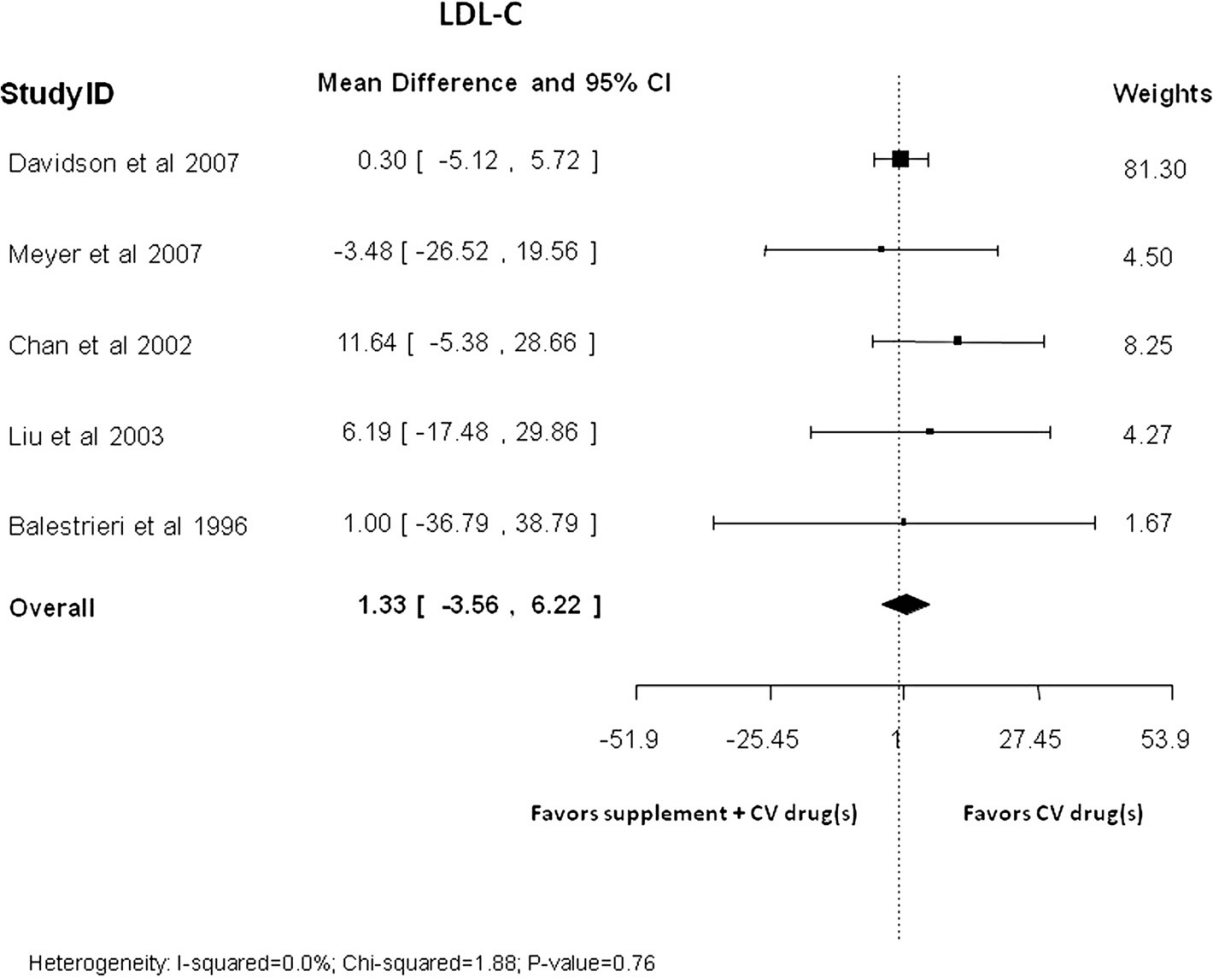
Omega-3 fatty acids co-administration with statins versus statins alone: post-treatment low density lipoprotein-cholesterol levels.

**Figure 3:**
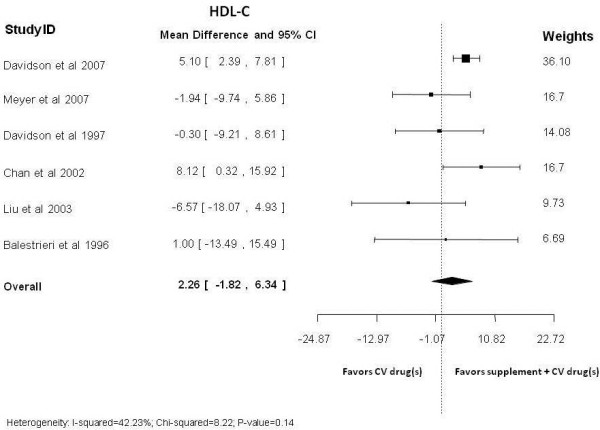
Omega-3 fatty acids co-administration with statins versus statins alone: post-treatment high density lipoprotein-cholesterol levels.

Co-administration with statins showed no benefit of lowering low density lipoprotein-cholesterol (LDL-C) (Figure 2) [41,43,52,74,81], total cholesterol [41,43,52,74,81], or of increasing HDL-C (grade: low) (Figure 3) [41,43,52,74,75,81]. However, the effect of adding statins to omega-3 fatty acids in participants with high baseline triglyceride levels (>200 mg/mL) was beneficial in lowering post-treatment triglyceride levels [41,73] (grade: low), as opposed to participants with lower baseline levels of triglyceride for whom the results were inconclusive (grade: insufficient) [41,43,52,73,74,81]. Significant benefits in reducing systolic blood pressure (grade: low) [38,76], but no difference in diastolic blood pressure (grade: low) [74,76] were observed (Table 2).

#### ***Harms***

Insufficient evidence was available from 22 studies (21 RCTs and one retrospective cohort study) which examined omega-3 fatty acids plus statins [37,38,41,52,72,76,81-83,96,103], aspirin [46,51,77,80,84], aspirin and clopidogrel [99], aspirin in combination with dipyridamole and calcium channel blockers [51], warfarin [46,54], ramipril and/or irbesartan [78], or fenofibrate [55]. These studies were generally small and underpowered. They recruited healthy participants or participants with CHD or risk factors for CHD.

Meta-analyses of studies comparing omega-3 fatty acids and statin combinations versus statins alone yielded inconclusive results for serious adverse events (two studies; RR, 3.64 (95% CI 0.8, 17.2)), withdrawal due to adverse events (seven studies; RR, 1.2 (95% CI 0.6, 2.3)), elevated aspartate aminotransferase (two studies; RR, 0.6 (95% CI 0.3, 1.3)) and elevated alanine transaminase (four studies; RR, 0.9 (95% CI 0.5, 1.9)) (grade: insufficient) [37,38,41,72,81,83,96]. Non-significant and imprecise pooled estimates were also obtained for total adverse events and elevated creatine kinase (grade: insufficient). Forest plots are available online [100].

#### ***Pharmacokinetic outcomes***

In three open-label RCTs of healthy adult volunteers (sample size range 24 to 50) [37,83,96] taking 4 g/day omega-3 fatty acids and rosuvastatin, atorvastatin, 2-hydroxy atorvastatin, or 4-hydroxy atorvastatin, no statistically significant or clinically important differences were observed between the treatment and control groups for steady state AUC and C_max_ GMRs (grade: low). Observed changes in steady state β-hydroxysimvastatin arithmetic means of AUC, C_max_, t_max_clearance and half-life were not statistically significant (grade: insufficient).

### **Vitamin E**

#### ***Clinical outcome efficacy***

The evidence for the effect of vitamin E plus aspirin on stroke and transient ischemic attacks in selected patients with previous neurologic deficit was inconclusive (grade: insufficient) [48]. In one pragmatic trial, 19,934 women were randomized to vitamin E (600 IU/day) plus aspirin (100 mg/day) or aspirin alone for 10 years. No significant differences were noted for the composite outcome of nonfatal myocardial infarction, nonfatal stroke or vascular death (RR 0.95, 95% CI 0.79, 1.13) [40]. Although components of the composite outcome were gradable, shifts in incidence of stroke and heart attack might have been obscured in this composite outcome. No differences in adherence to statins were noted with co-administration of vitamin E.

#### ***Intermediate outcome efficacy***

In one study of 30 elderly patients at high risk of CHD, addition of vitamin E to nifedipine significantly lowered total cholesterol, LDL-C (grade: low), triglycerides (grade: low) and systolic blood pressure (inconclusive, grade: insufficient) [94] (Tables 2, 3).

#### ***Harms***

Evidence from 10 RCTs for co-administration of vitamin E and aspirin, nifedipine, furosemide, or statins in participants who were healthy, or who had CHD or risk factors for CHD was inconclusive (grade: insufficient). Sample sizes were generally small, except for one study that recruited over 9,000 women [40].

#### ***Pharmacokinetic outcomes***

No evidence.

### **Limitations of the results from the evidence**

Across all combinations of dietary supplements and CV drugs, the strength of evidence of the gradable outcomes of comparative efficacy or effectiveness was mostly graded as insufficient. Type II errors could not be excluded due to the low statistical power of mostly short-term efficacy trials, particularly with strict inclusion criteria excluding patients with uncontrolled comorbidities and acute ischemic events. In addition, most studies were short-term efficacy trials and thus unable to evaluate longer term effects from co-administration.

Limited findings on intermediate outcomes were available and the majority of evidence was contributed by small RCTs with statistically non-significant results and broad CIs. This imprecision precluded ruling in or out important benefits or harms, thus the strength of evidence for several gradable intermediate outcomes was rated insufficient. Statistically significant effects were graded as low strength of evidence because of limitations in the internal validity of studies, surrogacy of outcomes, and often absent reproducibility in the direction of effect estimates (Table 2). Forest plots are available online [100].

With respect to harms data, for all supplement-drug combinations examined, the strength of evidence from single or heterogeneous studies for bleeding, serious adverse events, withdrawal due to adverse events, renal dysfunction, hepatotoxicity and prolongation of corrected QT interval was insufficient. This was due to either inconsistent effect estimates across studies (suggesting conflicting findings with no obvious explanation) or imprecise estimates (precluding ruling out important benefit or harms in underpowered studies with wide confidence intervals).

## **Discussion**

Evidence gleaned from an initial search yielding over 32,000 records identified a considerable knowledge gap regarding the safety and efficacy of combining dietary supplements with CV drugs.

Among 168 records that addressed the relevant possibility of interaction, 63 studies contributed evidence for synthesis. With a few exceptions, there was insufficient evidence to draw any conclusions on particular interactions. In addition to an overall lack of evidence, the included studies were often underpowered to assess the predetermined clinically relevant outcomes set for this synthesis review. As well, many studies had important methodological limitations or were poorly generalizable to the relevant population. The strength of the identified evidence was frequently compromised by poor allocation concealment, and issues related to blinding, study reporting and potential conflict of interest. Drug interactions resulting in positive or negative outcomes likely occur, but the evidence available and identified in this review is insufficient to allow meaningful conclusions with confidence.

Available evidence comes primarily from short-term trials of highly selected participants, with limited external validity. The strength of evidence was low at best, with poor grading resulting from risks of bias, small sample sizes, and the fact that evidence is largely generated from intermediate surrogate outcomes rather than primary clinical endpoints. While there are data from which we can derive a sense of lack of interactions in some cases, the small size of the trials made it difficult to impossible to ascertain the potential for true clinical interaction.

Much of the pharmacokinetic research was conducted on healthy young adults; thus this evidence may not be applicable to populations with CVD and particularly for older patients taking CV drugs, due to possible differences in metabolism and the existence of comorbidities. The internal validity of most trials was compromised by flawed design, lack of appropriate allocation concealment and risk of bias. A formal assessment of statistical interaction was rarely undertaken. In the absence of corroborating pharmacokinetic evidence or assessment for statistical interaction, it is often impossible to determine whether a difference in outcome is due to true pharmacological interaction, or due to more independent additive, or possibly counteracting therapeutic effects.

A principal limitation of the included trials was that they were small and susceptible to type II errors. A marginally reassuring corollary is that if there was a real, dramatic clinical impact due to an interaction then some clinical effect would likely have been evident despite being underpowered for smaller effect sizes. Of more concern are possible interactions that could arise through polypharmacy of prescription drugs, a situation all too common, in particular for the elderly population.

With these caveats in mind, the following is a summary of the clearer signals from the evidence reviewed.

Omega-3 fatty acids (2 to 4 g/day) from fish and/or supplements likely do not interfere with the efficacy of statin therapy or calcium channel blockers in the presence of antiplatelet agents, and may provide an independent benefit in resolving hypertriglyceridemia. Also, garlic (4 to 10g/day) may not interact negatively with nitrates and warfarin and may confer independent benefit in improving HDL-C.

Interpretation of this report requires the reader to recognize that dietary supplements in the US are not regulated in the same manner as prescription drugs nor are manufacturers of dietary supplements held to the same standards with respect to providing evidence of efficacy and safety prior to marketing. Dietary supplements do not require FDA approval, nor are there any FDA regulations that require evidence of purity, quality or composition prior to marketing. This has resulted in a lack of standardization among products both from a single manufacturer and between manufacturers [104]. The lack of manufacturing regulation and labeling standards may result in significant differences between products, unbeknownst to the consumer, thereby limiting the external validity of clinical trials. Furthermore, there is little reliable published information regarding the safety of dietary supplements. Until only recently, manufacturers of dietary supplements were not obliged to report serious adverse events. This is quite different from what is required of prescription drugs.

Recent systematic reviews related to the topic of dietary supplement-drug interactions do not address the same scope, are not comprehensive, do not grade the outcomes extracted, do not evaluate the quality of evidence, or involve different populations of interest. A 2005 systematic review by Mills *et al.* focused on effects of natural health products on the metabolism of a broad range of conventional medicines [105]. Consistent with our review, they identified a lack of evidence supporting interactions between coenzyme Q10 and warfarin, *Ginkgo biloba* and warfarin, and *Ginkgo biloba* and digoxin. Both reviews also identified a shift in AUC of the international normalized ratio when American ginseng was taken in conjunction with warfarin; however, we question the clinical significance of this finding.

Also in 2005, Desai *et al.* reviewed interactions between dietary supplements and antiplatelet agents reported in clinical trials and case reports [106]. With respect to the supplements considered in our review, Desai concluded that omega-3 fatty acids along with aspirin led to significantly greater reductions in adenosine diphosphate-induced platelet aggregation, blood platelet count, thromboxane B2 and restenosis rates, as well as prolonged bleeding time. Vitamin E along with aspirin led to significantly greater reductions in platelet adhesion, ischemic stroke, recurrent episodes of transient ischemic attack, as well as prolonged dental bleeding time.

Reviews published by Izzo *et al.* in 2005 and Skalli *et al.* in 2007 described evidence for drug-supplement interactions, but the majority of evidence was generated from case reports and small case series [107,108]. The most recent systematic review on dietary supplement-drug interactions, published in 2010 by Kennedy and Seely, examined herb-drug interactions identified from trials wherein the herbal impact on hepatic metabolism via cytochrome P450 isoenzymes was ascertained [109]. Their target population was not specifically patients with CVD or CV drugs, and the review evaluated indirect evidence limited to herbs metabolized via the cytochrome P450 system. While some of the findings within these reviews are consistent with ours, our graded evaluation did not yield similar confidence in conclusions after evaluating the quality and strength of evidence.

A most obvious limitation of our review arose from the need to scope the work to a manageable yet relevant synthesis by restricting the number of dietary supplements to the sixteen that were considered. This subset of supplements was based on published North American usage surveys and a consensus selection process that included a Technical Experts Panel [6,21-26]. Thus, we omitted drugs and supplements, and related data from other international sources. The review also did not consider combinations of multiple dietary supplements with CV drugs to make causal inference possible. This limitation has negative implications for external validity because people with CVD likely self-prescribe combinations of numerous dietary supplements, taken alongside their CV drugs.

The greatest strengths of the present review are its broad search strategy and consideration of extensive literature, and a methodology that focused on the highest quality of evidence. We followed published guidance to pool data from studies, grade the strength of evidence, and assess applicability. Based upon preliminary searches we did not exclude German language literature, a language where most negative studies are published [110]. In order to minimize over-generalization of evidence, we also included only studies with more than 80% of participants taking each CV medication under consideration.

While a plethora of strong recommendations was the hope, we revealed instead a glaring knowledge gap regarding interactions between some of the agents most commonly used for their presumed pharmacological effects for serious chronic disease.

## **Conclusion**

With the continuing and increasing burden of CVD, precise recommendations are needed to guide the use of dietary supplements in disease prevention and management. An ungrounded call for avoidance of dietary supplements in conjunction with pharmaceutical treatments may result in the avoidance of potentially beneficial supplements, or just as importantly might be readily ignored, leading to potential harm with negative interactions. Strategic investment to build research capacity to address the important knowledge gap in the area of dietary supplement-drug interactions should address relevant questions in appropriate populations according to disease, genetic makeup, age, and so on. While awaiting results of future, adequately powered interventional trials focusing on meaningful clinical outcomes, evidence from well-conducted prospective observational studies should be sought. Electronic health record linkages between databases of dietary supplement use and CV drug prescription may also add to the equipoise that has been so insufficiently addressed to date.

## **Abbreviations**

AHRQ: Agency for Healthcare Research and Quality; AUC: area under the curve; CI: confidence interval; CHD: coronary heart disease; CV: cardiovascular; CVD: cardiovascular disease; FDA: Food and Drug Administration; GMR: geometric mean ratio; HDL-C: high density lipoprotein-cholesterol; INR: International Normalized Ratio; LDL-C: low density lipoprotein-cholesterol; MD: mean difference; OR: odds ratios; RCT: randomized clinical trials; RR: relative risk.

## **Competing interests**

The authors declare that they have no competing interests.

## **Authors’ contribution**

SK and DS were involved in the conception and design, data interpretation, reviewing and revising the manuscript critically for important intellectual content, and its final approval. FY, KS, AT, ACT and MS analyzed and interpreted data, reviewed the draft and revised it critically for important intellectual content, and approved the manuscript. JT was involved in the conception and design, reviewed the draft and revised the manuscript critically for important intellectual content, and approved the manuscript. TCO, MAT and BS were involved in the conception and design, reviewed the draft and revised it critically for important intellectual content, and approved the manuscript. MTA was involved in the conception and design, data analysis and interpretation, drafting of the manuscript, and final approval of the manuscript. All authors read and approved the final manuscript.

## *Disclaimer*

This project was funded under Contract No. HHSA290-2007-10059-I (EPCIII) from the AHRQ, US Department of Health and Human Services. The authors of this report are responsible for its content. Statements in the report should not be construed as endorsement by the AHRQ, National Institute of Health or the US Department of Health and Human Services.

## Supplementary Material

Additional file 1 **Table S1. **Selected cardiovascular drugs for current review. Click here for file

Additional file 2 **Table S2. **List of included outcomes. Click here for file

Additional file 3 **Table S3. ***a Priori *outcomes for grading the strength of evidence. Click here for file
