# Supplementary material for: Interactions of commonly used dietary supplements with cardiovascular drugs: a systematic review
Source: Syst Rev. 2012 May 31;1:26. doi: 10.1186/2046-4053-1-26 (PMC3534595; doi:10.1186/2046-4053-1-26)
Supplement: Additional file 1 — Table S1. Selected cardiovascular drugs for current review. [file 2046-4053-1-26-S1.doc]

Additional file 1: Appendix **Table S1.** **Selected cardiovascular drugs for current review**

| **Cardiovascular Drug Category** | **Drug Class** | **Drugs of Interest** |
| --- | --- | --- |
| Alpha adrenergic blockers |  | Doxazosin; Prazosin; Terazosin |
| Antiarrhythmic drugs | Class Ia | Disopyramide; Procainamide; Quinidine |
|  | Class Ib | Mexilitine |
|  | Class Ic | Encainide; Flecainide; Propafenone |
|  | Class III | Amiodarone; Dofetilide; Dronedarone |
| Anticoagulants | Coumarin derivatives | Warfarin |
|  | Heparins | Dalteparin; Enoxaparin; Heparin;Tinzaparin |
| Antilipemic agents | Bile acid sequestrants | Colestipol colesevelam Cholestyramine resin |
|  | Cholesterol absorption inhibitor | Ezetimibe |
|  | Fibric acid Derivatives | Fenofibrate; Gemfibrozil |
|  | HMG-CoA reductase Inhibitors | Atorvastatin; Fluvastatin; Lovastatin; Pravastatin; Rosuvastatin; Simvastatin |
| Antiplatelet agents |  | Acetylsalicylic acid (ASA); Clopidogrel ticlopidine |
| Beta adrenergic antagonists |  | Acebutalol; Betaxolol; Bisoprolol; Carvedilol;Labetalol; Metoprolol; Nadolol; Nebivolol; Pindolol; ropranolol; Sotalol; Timolol |
| Calcium channel antagonists | Dihydropyridine | Amlodipine; Felodipine; Isradipine; Nicardipine; Nifedipine; Nimodipine; Nisoldipine |
|  | Non-Dihydropyridine | Diltiazem; Verapamil |
| Diuretics | Loop diuretics | Bumetanide; Ethacrynic acid; Furosemide; Torsemide |
|  | potassium-sparing (K-sparing) | Amilioride; Triamterene |
|  | Thiazide | Bendroflumethazide; Chlorothiazide;Hydrochlorothiazide; Methyclothiazide; Polythiazide |
|  | Thiazide-like | Chlorthalidone; Indapamide; Metolazone |
| Inotropic agents |  | Digoxin |
| Renin-angiotensin-aldosterone system antagonists | Angiotensin-converting enzyme inhibitors (ACE inhibitors) | Benazepril; Captopril; Enalapril; Fosinopril; Lisinopril; Moexipril; Perindopril; Quinapril; Ramipril; Trandolapril |
|  | Angiotensin II receptor blockers (ARBs) | Candesartan; Eprosartan; Irbesartan; Losartan; Olmesartan; Telmisartan; Valsartan |
|  | Renin inhibitors | Aliskiren |
|  | Aldosterone receptor antagonist | Eplerenone; Spironolactone |
| Vasodilators | Central vasodilators | Clonidine; Guanabenz; Guanfacine; Methyldopa |
|  | Direct vasodilators | Diazoxide; Hydralazine; Minoxidil |
|  | Nitrates | Isosorbide dinitrate |
|  |  | Nitroglycerin |
|  | Phosphodiesterase inhibitors | Cilostazol; Sildenafil; Tadalafil; Vardenafil; |
|  | Prostacycline | Alprostadil; Epoprostenol; Iloprost; Treprostinil |
|  | Endothelin antagonist | Ambrisentan; Bosentan |
|  | Miscellaneous vasodilators | Papaverine; Isoxsuprine; Rauwolfia alkaloids |
