# Supplementary material for: Interactions of commonly used dietary supplements with cardiovascular drugs: a systematic review
Source: Syst Rev. 2012 May 31;1:26. doi: 10.1186/2046-4053-1-26 (PMC3534595; doi:10.1186/2046-4053-1-26)
Supplement: Additional file 2 — Table S2. List of included outcomes. [file 2046-4053-1-26-S2.doc]

Additional file 2: Appendix Table S2**.** **List of included outcomes**

| **Outcome Category** | **Outcome** |
| --- | --- |
| **Clinical outcomes** | Mortality |
|  | **•** All-cause mortality |
|  | **•** Vascular death |
|  | **•** Specific vascular death (e.g., fatal MI, fatal stroke) |
|  | Ischemic Heart Disease (Coronary Artery Disease) |
|  | **•** All myocardial infarction (MI, acute MI) |
|  | **•** Nonfatal MI (MI, acute MI) |
|  | **•** Unstable angina |
|  | **•** Acute coronary syndrome |
|  | **•** Coronary artery disease composite outcomes (combination) |
|  | **•** Coronary (re)stenosis/graft occlusion/vasospasm (*post hoc*) |
|  | Arrhythmias |
|  | **•** Sudden death |
|  | **•** Ventricular fibrillation |
|  | **•** Ventricular tachycardia |
|  | **•** Atrial fibrillation |
|  | **•** Heart block |
|  | Other heart disease |
|  | **•** Congestive heart failure |
|  | **•** Valvular disease |
|  | Adherence to prescribed cardiovascular drug or regimen |
|  | Hospitalization |
|  | Cerebrovascular disease |
|  | **•** All stroke |
|  | **•** Hemorrhagic stroke |
|  | **•** Thrombotic stroke |
|  | **•** Transient ischemia attack (TIA) |
|  | **•** Carotid artery disease (not measured by IMT or Doppler) |
|  | **•** Other |
|  | Peripheral arterial disease (PAD) |
|  | **•** Limb thrombosis/leg ischemia |
|  | **•** Claudication (pain walking) |
|  | **•** Mesenteric ischemia |
|  | **•** Abdominal aortic aneurysm |
|  | **•** Ankle-brachial index |
|  | **•** Other reported clinical PAD |
|  | CVD surgery and procedures |
|  | **•** Coronary artery revascularization (coronary artery bypass graft [CABG], percutaneous transluminal coronary angioplasty [PTCA], stent) |
|  | **•** Valve replacement |
|  | **•** Carotid revascularization (± stent) |
|  | **•** Peripheral revascularization (± stent) |
|  | **•** Amputation |
|  | Syncope |
|  | Quality of life |
|  | Renal replacement therapy |
| **Intermediate outcomes (limited to established outcomes)** | Lipids |
|  | **•** Total cholesterol |
|  | **•** Low-density lipoprotein cholesterol (LDL-C) |
|  | **•** High-density lipoprotein cholesterol (HDL-C) |
|  | **•** Triglycerides |
|  | **•** Lipoprotein a (LP(a)) |
|  | **•** Non–HDL-C |
|  | Other serum markers |
|  | **•** C-reactive protein (CRP) |
|  | Blood pressure |
|  | **•** Systolic (SBP) |
|  | **•** Diastolic (DBP) |
|  | **•** Hypertension (HTN), new or worsening (e.g., need for change in therapy) |
|  | **•** Hypotension |
|  | Electrocardiographic (ECG) measurements (≥24 h Holter monitor, PR interval) other than established arrhythmias (based on context, this might be evaluated as a harms outcome rather than efficacy) |
|  | Other diagnostic tests |
|  | **•** Carotid intima-media thickness (IMT), as measured by Doppler ultrasound |
|  | **•** Coronary/cerebral arterial calcification |
|  | Platelet aggregability |
|  | Bleeding and coagulation times |
|  | Ejection fraction |
|  | Incidence of metabolic syndrome and change in 10-year Framingham risk profile |
| **Harms** | Clinical adverse events |
|  | **•** Serious adverse events (composite outcome)^c^ |
|  | **•** Neurologic adverse events (e.g., neuropathy, seizure) |
|  | **•** Allergic reactions (e.g., anaphylaxis, skin, transient acute airway disease) |
|  | **•** Gastrointestinal adverse events (e.g., diarrhea, constipation, nausea/vomiting) |
|  | **•** Clinically significant bleeding (e.g., intracerebral/intraventricular, gastrointestinal, hematuria) |
|  | **•** Withdrawal due to adverse events |
|  | **•** Other reported important clinical adverse events |
|  | Organ toxicity |
|  | **•** Liver (aspartate aminotransferase [AST], alanine aminotransferase [ALT], alkaline phosphatase [ALP], or hepatitis) |
|  | **•** Renal (blood urea nitrogen [BUN], creatinine, glomerular filtration rate [GFR]) |
|  | **•** Bone marrow (e.g., leukopenia, anemia, neutropenia, thrombocytopenia) |
|  | Fasting blood glucose |
|  | Hemoglobin A_1c_ |
|  | QT interval |
|  | Other harms |
| **Pharmacokinetic** | Absorption |
|  | **•** Bioavailability (F), maximum drug concentration (C_max_) and time to C_max_ (t_max_) (latter two were added post hoc) |
|  | Area under the concentration curve (AUC) |
|  | Distribution |
|  | **•** Volume of distribution (Vd; e.g., in L/kg) |
|  | Metabolism/Excretion |
|  | Clearance, elimination constant (K_el_), half life (t_1/2_) |
