# Supplementary material for: Interactions of commonly used dietary supplements with cardiovascular drugs: a systematic review
Source: Syst Rev. 2012 May 31;1:26. doi: 10.1186/2046-4053-1-26 (PMC3534595; doi:10.1186/2046-4053-1-26)
Supplement: Additional file 3 — Table S3. a Priori outcomes for grading the strength of evidence. [file 2046-4053-1-26-S3.doc]

Additional file 3: Appendix Table S**3.** ***a Priori*** **outcomes for grading the strength of evidence**

| **Category** | **Outcomes** |
| --- | --- |
| Clinical efficacy/effectiveness |  |
|  | • Mortality (all-cause and vascular death) |
|  | • Myocardial ischemic events (fatal myocardial infarction, nonfatal myocardial infarction, unspecified myocardial infarction, and acute coronary syndromes) |
|  | • Cerebrovascular events (hemorrhagic/ischemic/unspecified stroke) |
|  | • Hospitalization |
|  | • Arrhythmia |
|  | • Clinical outcomes of peripheral arterial disease |
|  | • Quality of life |
| Intermediate efficacy | • Blood pressure (systolic and diastolic) |
|  | • Lipid profile (low density lipoprotein, high density lipoprotein, non–high density lipoprotein cholesterol and triglycerides) |
|  | • International normalised ratio (INR) for coumarin derivatives |
|  | • Incidence of metabolic syndrome |
|  | • Change in 10-year Framingham risk profile |
| Harms | • Serious adverse events (composite outcome according to the Food and Drug Administration definition of serious adverse events),(1) |
|  | • Withdrawal due to adverse events |
|  | • Clinical bleeding (intracranial, gastrointestinal, genitourinary, subretinal, etc.) |
|  | • Renal dysfunction (e.g., proteinuria, elevated creatinine, need for transplant, glomerular filtration rate) |
|  | • Hepatotoxicity (elevated enzymes or fulminant failure) |
|  | • QT prolongation. |
| Pharmacokinetic measures | • Area under the plasma cardiovascular drug concentration-time curve (AUC) |
|  | • Maximum drug concentration (C_max_) |
|  | • Drug half-life (t_1/2_) |
|  | • Drug clearance |
